# Two novel variants of *VPS13C* gene related Parkinsonism: A case report and literature review

**DOI:** 10.1097/MD.0000000000047063

**Published:** 2026-01-09

**Authors:** Yanyan Jiang, Chenghe Fan

**Affiliations:** a Department of Neurology, The First Affiliated Hospital of Zhengzhou University, Zhengzhou, Henan Province, China.

**Keywords:** dementia with Lewy bodies, magnetic resonance imaging, Parkinson’s disease, Parkinsonism, *VPS13C*

## Abstract

**Rationale:**

Mutations in the vacuolar protein sorting 13 homolog C (*VPS13C*) gene have been associated with Parkinson disease (PD). However, the mutation of *VPS13C* in Parkinsonism is uncommon and the clinical characteristics are highly heterogeneous. This study identifies 2 novel pathogenic variants in *VPS13C*, with particular emphasis on follow-up brain magnetic resonance imaging (MRI) images.

**Patient concerns:**

The patient first exhibited resting tremor in the right limb at the age of 26. As the disease progressed, he developed bradykinesia, rigidity, gait instability, cognitive decline, dysarthria, myoclonus, depressed mood, irritability, and aggression. He was treated with levodopa/benserazide, pramipexole, and rasagiline, but the benefit was only temporary. By the age of 32, his gait instability was further aggravated, manifested as frequent falls, requiring bed rest or wheelchair use. Follow-up brain MRI showed progressive cortical atrophy. Whole-exome sequencing of the patient revealed compound heterozygous pathogenic variants (c.1699C > T, chr15: 62156504–62352664) in *VPS13C*.

**Diagnoses:**

*VPS13C*-related early onset PD.

**Interventions:**

The patient was treated with levodopa/benserazide 125 mg 4 times daily, rasagiline 1 mg once daily, donepezil 5 mg once nightly, and quetiapine 50 mg once nightly.

**Outcomes:**

After 6 months of follow-up, his symptoms were further aggravated.

**Lessons:**

This study identifies 2 novel pathogenic variants in *VPS13C*, expanding the known mutational spectrum of the gene. Additionally, brain MRI may serve as a potential imaging marker for disease progression. A review of the literature indicates that *VPS13C*-related Parkinsonism appears as a heterogeneous disorder, including PD and dementia with Lewy bodies. *VPS13C* mutations are highly diverse, with point mutations being the most common, followed by splice-site variants. Genetic screening is essential for an accurate diagnosis and distinction between different forms of early onset PD. This increases clinicians’ understanding of the clinical and genetic characteristics of *VPS13C*-related Parkinsonism.

## 1. Introduction

Early-onset Parkinson disease (EOPD) is a neurodegenerative disease associated with several genetic factors. Compound heterozygous and homozygous mutations in the vacuolar protein sorting 13 homolog C (*VPS13C*) gene were described as a rare cause of autosomal-recessive EOPD.^[1,2]^ Additionally, *VPS13C,* previously linked to sporadic PD in genome-wide association studies, has been implicated in disease development.^[3]^ The advancement of PD is linked to several mechanisms, including misfolding and aggregation of α-synuclein, neuroinflammation, malfunctioning in protein clearance systems, mitochondrial dysfunction, ubiquitin-mediated proteasome system, and autophagy-mediated lysosome system.^[4]^ However, the mechanism by which *VPS13C* loss of function leads to PD remains unclear. VPS13 is the founding member of a family of proteins that mediate lipid transfer at intracellular membrane contact sites by a bridge-like mechanism. Mammalian genomes comprise 4 *VPS13* genes (*VPS13A*, *VPS13B*, *VPS13C*, and *VPS13D*) encoding proteins with distinct localizations and function. In humans, the absence of VPS13A and VPS13C proteins does not affect early life but leads to age-related neurodegenerative diseases.^[5]^ *VPS13C* spans 208 kb and contains 86 exons encoding a 3753-amino acid protein with a chorein domain at its N terminus, a DUF1162 domain of unknown function, and a putative autophagy-related domain.^[1]^ Earlier studies identified *VPS13C* on the mitochondrial surface and demonstrated its influence on mitochondrial activity and maintenance as well as multiple interdependent loops between *parkin, PINK1*, and *VPS13C* in the regulation of mitophagy.^[1,6]^ These findings further highlight the role of *VPS13C* in mitochondrial dysfunction-pathological processes intricately linked to α-synuclein biology in PD pathogenesis. However, subsequent studies showed that *VPS13C* encodes a lipid transfer protein localized at contact sites between the endoplasmic reticulum and late endosomes/lysosomes.^[7,8]^ Recent findings suggest that *VPS13C* contributes to PD pathogenesis through dysregulation of several lysosomal pathways.^[9]^ Lipid transport mediated by VPS13C via a bridge-like mechanism may play a role in preventing or repairing the rupture of the lysosomal membrane. For this reason, loss-of-function mutations in *VPS13C* may contribute to the development of PD.^[10]^ Despite many remaining unanswered questions, we now know of 2 major interconnected cellular pathways that are altered and involved in PD pathogenesis: lysosomal function, and mitochondrial maintenance. Therefore, *VPS13C* might play an important role in the pathogenesis of PD via the mitochondria and lysosomal function pathway. Understanding the contribution of the different mutated *VPS13C* alleles to the genetic etiology needs additional research.

The mutation of *VPS13C* in Parkinsonism is uncommon.^[11]^ Besides, the clinical manifestations of *VPS13C*-related Parkinsonism are highly heterogeneous. The main clinical manifestations are severe disease progression and the co-occurrence of Parkinsonism and dementia. Originally reported in 2016, bi-allelic truncating variants in this gene were described as a very rare cause of rapidly progressing EOPD with early cognitive decline.^[1]^ Subsequently, a case of early-onset Parkinsonism with a large homozygous *VPS13C* deletion was reported. It is worth noting that the patient showed normal cognitive status and a milder clinical picture than that observed in previously reported patients.^[2]^ Interestingly, a Japanese study reported compound heterozygous nonsense variants in *VPS13C* in 2 siblings diagnosed with dementia with Lewy bodies (DLB) without obvious Parkinsonism.^[12]^ Overview of previously reported *VPS13C* variants indicates that some patients present with early-onset, rapidly progressive Parkinsonism, and early cognitive decline, whereas others exhibit late-onset, cognitive dysfunction, and psychiatric symptoms without obvious Parkinsonism. Furthermore, the types of genetic variants are highly heterogeneous, and a definitive genotype–phenotype correlation remains elusive. As more cases are documented, the relationship between genotype and phenotype is expected to become clearer. In this study, we present a case of 2 novel *VPS13C* variants and review the existing literature on the clinical and genetic features of *VPS13C*-associated neurodegeneration.

## 2. Case presentation

**Figure 1.**
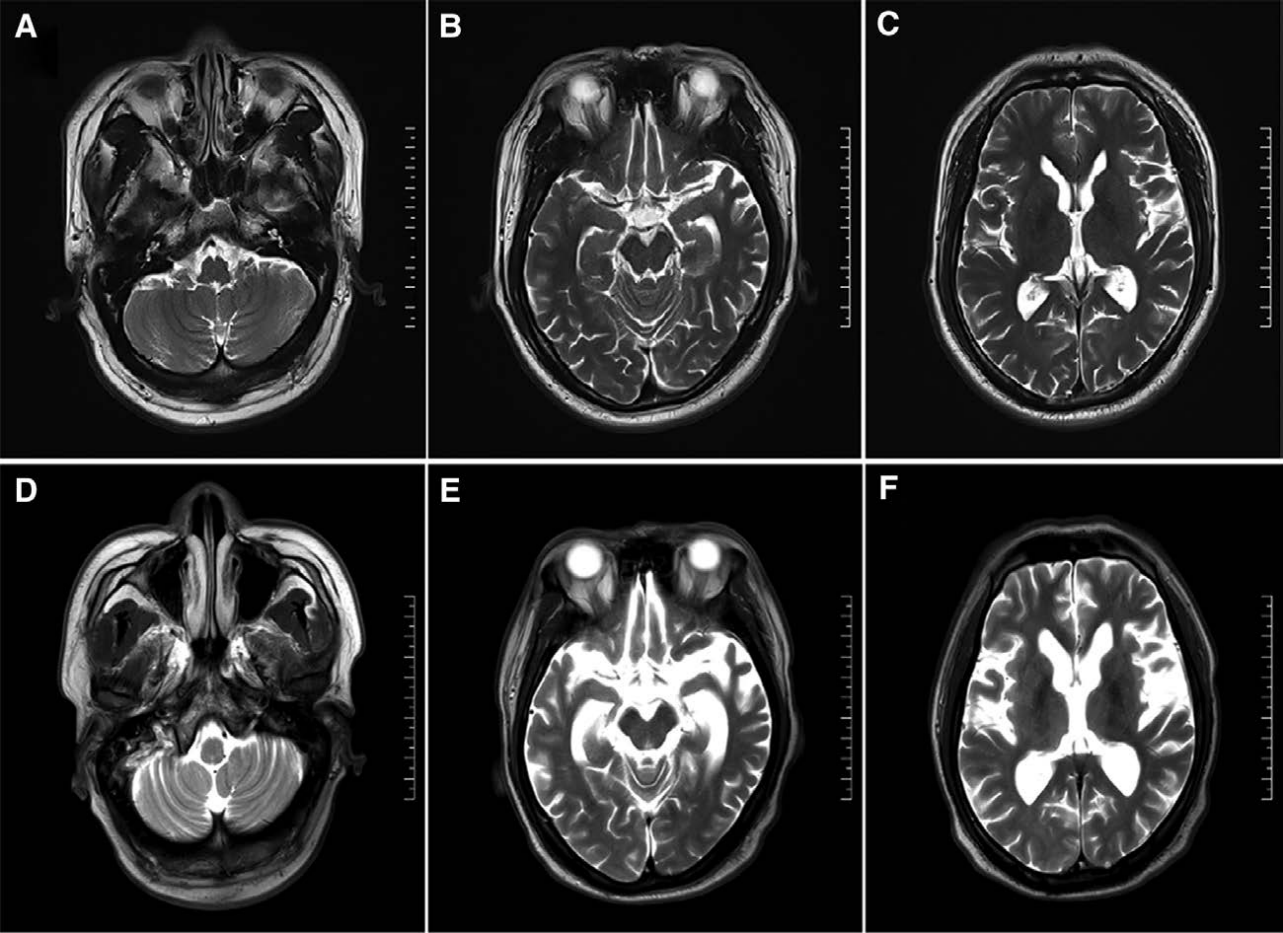
Brain magnetic resonance imaging (MRI) examination of patient. (A–C) T2 sequences demonstrated mild cortical atrophy at 2 years of onset. (D–F) T2 sequences demonstrated general brain atrophy, especially in lobes cortex and hippocampus at 6 years of onset. At 2 years post-onset, the Global Cortical Atrophy score was 1 bilaterally in the frontal lobes, increasing to 2 at 6 years; at 2 years, the score was 1 bilaterally in the temporal lobes, increasing to 3 at 6 years.

A 32-year-old Han Chinese male farmer presented to the hospital with resting tremor, severe movement disorder, gait instability, cognitive decline, dysarthria, urinary incontinence, and mental and behavioral disorders. At the age of 26, he presented with resting tremor and bradykinesia in the right limb. One year later, the resting tremor affected his left limb. He was initiated on levodopa, which provided good control of motor symptoms. By the age of 28, his symptoms had progressed, with worsening gait instability and mild cognitive decline. Brain magnetic resonance imaging (MRI) revealed only mild cortical atrophy (Figs. 1A-C). The Global Cortical Atrophy (GCA) score was 1 bilaterally in the frontal and temporal lobes. MRI data were acquired using 3.0-Tesla Magnetom Trio Tim MR scanner (Siemens, Erlangen, Germany). The patient was subjected to conventional MRI protocols, including T1-weighted imaging, T2-weighted imaging, fluid-attenuated inversion recovery, diffusion-weighted imaging, and susceptibility weighted imaging. T2-weighted axial images were used to calculate the GCA scale. Cortical atrophy was assessed with the GCA scale (range 0–3; 0 = no atrophy, 1 = mild, 2 = moderate, 3 = severe).^[13]^ At 29 years old, the patient developed depression, anxiety, obsession, severe psychomotor agitation, and violent behaviors. He was treated with levodopa/benserazide, and rasagiline, but the benefit was only temporary. By the age of 32, he developed further cognitive deterioration, and his gait instability was further aggravated, manifested as frequent falls, requiring bed rest or wheelchair use. In addition, he developed dysarthria, urinary incontinence, and myoclonus. However, he did not exhibit orthostatic hypotension, hyposmia, or rapid eye movement sleep behavior disorder. He was the second child of non-consanguineous healthy parents with no family history of neurological disorders. The patient’s older sister was in good health.

Neurological examination revealed severe dysarthria. His orientation, memory, calculation, and comprehension were impaired. He showed limited upward vertical gaze and rigidity in all limbs and the trunk. The muscle strength remained normal. The patient was unable to complete the bilateral heel-knee-shin test. The rest tremor and myoclonus of both limbs were observed. His gait was unsteady, wide-based, and slow. The pull test was positive. Tendon reflexes were brisk in both upper limbs and active in both lower limbs. The bilateral Babinski sign and meningeal irritation signs were negative. His Movement Disorder Society-Unified Parkinson Disease Rating Scale score was 89, and the Mini-Mental State Examination score was reduced (7/30).

The results of the auxiliary examination suggested that there were no abnormalities in blood count, urinalysis, routine stool, biochemical indicators, homocysteine, ceruloplasmin, hepatitis, syphilis, HIV, coagulation function, and residual urine volume. The serum level of superoxide dismutase-1 was 236 U/mL, which was higher than the upper limit of the normal range (110–215 U/mL). The interleukin-6 level was 1.8 pg/mL, which was within the normal range (0–7 pg/mL). Brain MRI indicated general brain atrophy, especially in the bilateral frontal, temporal, and parietal lobes, cortex, and hippocampus (Fig. 1D–F). The GCA score was 2 bilaterally in the frontal lobes and 3 bilaterally in the temporal lobes. Susceptibility-weighted imaging results were normal. Electroencephalography revealed a moderately diffuse slow wave. Lumbar puncture revealed normal cell counts, protein content, and autoimmune encephalitis antibodies.

**Figure 2.**
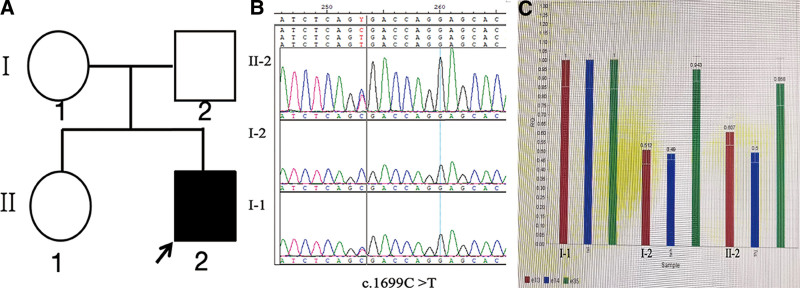
Family pedigrees and sequencing data. (A) The pedigree chart of family. (B) The Sanger sequence chromatogram of the patient and his parents. (C) Quantitative polymerase chain reaction confirmed large deletion variant of the patient and his parents.

The patient’s whole-exome sequence (WES) identified compound heterozygous variants in the *VPS13C* gene: a nonsense variant (c.1699C > T, p.Arg567*) and a large deletion variant (chr15: 62156504–62352664). The nonsense mutation was located on chr15:62277152, resulting in a nucleotide substitution from C to T, which created a premature stop codon and predicted a truncated protein. The presence of *VPS13C* exon deletions (exons13, 14, and 35) expanding the deletion breakpoints (chr15: 62156504–62352664) was confirmed by copy number variation quantification through quantitative polymerase chain reaction. Both variants have not been previously reported and were not found in the Thousand Human Genome Database. The nonsense variant c.1699C > T was predicted to be pathological based on the American College of medical genetics and Genomics criteria. The evidence for pathogenic includes 1 very strong evidence of pathogenicity (PVS1) and 2 moderate evidence of pathogenicity (PM2 and PM3) and 2 supporting evidence of pathogenicity (PP3 and PP4). The Mutation Taster software predicted it as a pathogenic variation, and the probability was 100%. The results of pedigree verification suggested that the proband’s mother was an asymptomatic carrier of the nonsense variant c.1699C > T of *VPS13C* gene, as assessed by Sanger sequencing, and proband’s father was an asymptomatic carrier with the exons deletion, as assessed by quantitative polymerase chain reaction (Fig. 2).

**Figure 3.**
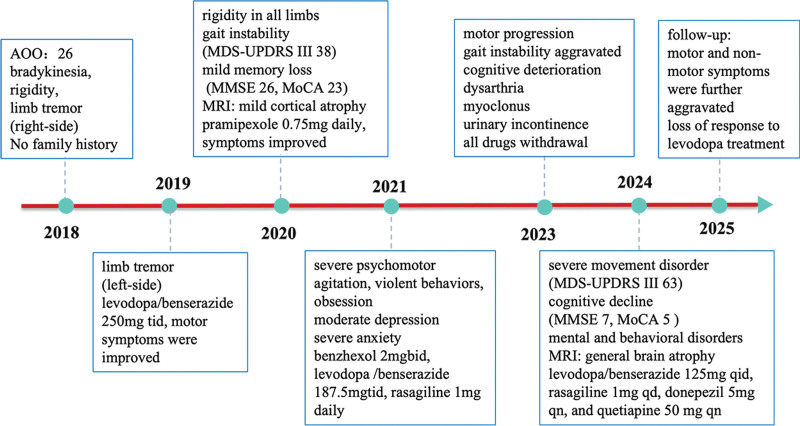
Clinical timeline of the patient. AAO = age at onset, MDS-UPDRS = movement disorder society-unified Parkinson’s disease rating scale, MMSE = mini-mental state examination, MoCA = Montreal Cognitive Assessment, MRI = magnetic resonance imaging.

The patient was diagnosed with *VPS13C*-related EOPD. He was treated with levodopa/benserazide 125 mg 4 times daily, rasagiline 1 mg once daily, donepezil 5 mg once nightly, and quetiapine 50 mg once nightly. After 6 months of follow-up, his symptoms were further aggravated. The patient’s clinical timeline was showed in Figure 3, which outlines key events and interventions in the patient’s care.

## 3. Discussion

Combining with clinical symptoms and WES, we diagnosed this patient as *VPS13C*-related EOPD. Given its atypical clinical phenotype, a differential diagnosis remains necessary. Monogenic typical Parkinsonism: The initial presentation – including early-onset Parkinsonism and a favorable response to levodopa – resembles that seen in EOPD patients with mutations in *PARK2*, *PINK1*, or *DJ-1*. However, the subsequent rapid progression – marked by worsening motor deficits, cognitive decline, and loss of levodopa response – along with the emergence of myoclonus and cortical atrophy, distinguishes this case from the classical monogenic EOPD. Age at onset, presence of specific signs, and degree of levodopa response inform differential diagnostic considerations and genetic testing indications in atypical forms of Parkinsonism.^[14]^ DLB: according to the DLB consortium criteria, a key diagnostic guideline is the timing of cognitive relative to motor symptoms. This patient initially presented with resting tremor and bradykinesia. Two years later, the patient developed mild cognitive impairment. The cognitive deficits were non-fluctuating and not accompanied by vivid visual hallucinations, which does not support a diagnosis of DLB. Frontotemporal dementia: the emergence of cognitive and psychiatric symptoms several years after the onset of motor symptoms, early age of onset, and positive initial response to levodopa therapy all argue against a diagnosis of frontotemporal dementia.

Therapeutic management of patients with *VPS13C*-related Parkinsonism poses significant challenges. Initially, the patient was treated with levodopa monotherapy during the early disease stage. However, as motor symptoms progressed, and the efficacy of levodopa declined after 1 year. Given the patient’s diagnosis of EOPD, the treatment was switched to pramipexole extended-release tablets. Two years later, the patient developed psychiatric symptoms. Given that pramipexole may exacerbate such symptoms, it was discontinued and replaced with a combination of levodopa/benserazide and rasagiline. Due to prominent tremors, a low dose of benzhexol was introduced. After another 2 years, the patient showed significant cognitive decline, prompting immediate discontinuation of benzhexol and the initiation of donepezil to improve cognitive function. Additionally, quetiapine was added to address the prominent psychiatric and behavioral abnormalities. Caution is warranted with the use of pramipexole and benzhexol in patients with *VPS13C*-related Parkinsonism, given their susceptibility to cognitive impairment and psychiatric symptoms.

To date, only 19 clinically described cases of *VPS13C*-related Parkinsonism have been reported in the literature.^[1,2,11,12,15–17]^ A summary of the demographic, clinical, genetic, and radiological data of these patients, along with the present case, is provided (Table 1). The clinical manifestations were very diverse, divided into the following 3 categories: classical clinical features of PD (bradykinesia, rigidity, rest tremor, freezing, postural instability, motor fluctuations, and dysautonomia), classical clinical features of DLB (visual hallucinations, cognitive decline, psychiatric symptoms, behavioral disorders, and myoclonus), and atypical features (dystonia, non-fluent aphasia, hearing impairment, supranuclear gaze palsy, and seizure). The clinical features of our case closely resemble those reported in previous studies of patients with the PD phenotype. In contrast, patients with the DLB phenotype had a relatively late age of onset, ranging from 41 to 62 years. There were significant differences in initial symptoms and clinical manifestations between patients with PD and those with the DLB phenotype. Patients with PD usually present with rigidity and resting tremors as initial symptoms. As the disease progresses rapidly, it may be accompanied by symptoms such as cognitive decline, dysautonomia, dysarthria, and mental and behavioral disorders. Patients with the DLB phenotype usually have hallucinations and language problems as initial symptoms. As the disease progresses, it may be accompanied by symptoms such as cognitive decline, psychiatric symptoms, behavioral disorders, and myoclonus. Few patients exhibit peculiar phenotypic findings, such as hearing impairment, oculomotor disturbances, apraxia, and seizures.^[16,17]^ Most patients responded to levodopa at early stages, but disease progression was particularly severe, with early cognitive decline, axial symptoms, and eventual loss of response to levodopa treatment.

**Table 1:** A retrospective analysis of *VPS13C*-related Parkinsonism literature has been reported.

Number	Family source	Sex	AAO	Initial symptom	Parkinsonism	Cognitive decline	Brain MRI	Variant1	Variant2	References
1	Turkish	F	<46	Depression, rigidity	+	+	Atrophy	c.8445.2T > G	c.8445.2T > G	Lesage et al^[1]^
2	French	M	33	Rigidity, tremor	+	+	Normal	c.806_807insCAGA	c.9568G > T	Lesage et al^[1]^
3	French	F	25	Rigidity, dystonia	+	+	Normal	c.4165G > C	c.4777delC	Lesage et al^[1]^
4	Caucasian	F	39	Rigidity, tremor	+	+	NA	c.2029 + 2T > G	c.3215-1G > T	Schormair et al^[10]^
5	Iranian	F	20	Bradykinesia, tremor	+	−	Normal	c.1353 + 3558_9106-7010del	c.1353 + 3558_9106-7010del	Darvish et al^[2]^
6	Chinese	M	32	Tremor	+	−	NA	c.6241A > T	c.2086C > A	Gu et al^[15]^
7	Chinese	F	41	Rigidity	+	NA	NA	c.6880C > T	c.1290.1G > A	Gu et al^[15]^
8	Chinese	F	18	Rigidity	+	+	NA	c.6554C > T	c.2771T > G	Gu et al^[15]^
9	Chinese	M	35	Tremor	+	−	NA	c.10237A > C	c.1291-4A > G	Gu et al^[15]^
10	Chinese	F	44	Rigidity	+	+	NA	c.1291-4A > G	c.809C > T	Gu et al^[15]^
11	Chinese	F	36	Tremor	+	−	NA	c.392A > G	c.11038C > T	Gu et al^[15]^
12	Chinese	F	36	Tremor	+	+	NA	c.1291-4A > G	c.6128C > G	Gu et al ^15]^
13	Japanese	F	48	Hallucinations	–	+	Atrophy	c.2755G *> *T	c.10522C *> *T	Kobayashi et al^[12]^
14	Japanese	F	62	Amnesia	–	+	Atrophy	c.2755G *> *T	c.10522C *> *T	Kobayashi et al ^12]^
15	Belgian	NA	42	Language problems	+	+	Atrophy	c.1185G > C	c.1330G > C	Smolders et al^[16]^
16	Belgian	NA	41	Language problems	+	+	Atrophy	c.1185G > C	c.1330G > C	Smolders et al^[16]^
17	NA	NA	58	Memory problems,	+	+	NA	c.3652A > G	c.8366 T > C	Smolders et al^[16]^
18	Italian	F	42	Bradykinesia	+	+	Atrophy	c.860_866dupATATACC	c.860_866dupATATACC	Monfrini et al^[17]^
19	Italian	F	43	Dystonia	+	+	Atrophy	c.532delA	c.7806C* > *G and c.4669G > C	Monfrini et al^[17]^
20	Chinese	M	26	Tremor	+	+	Atrophy	c.1699C > T	chr15: 62156504-62352664	This case

AAO = age at onset, F = female, M = male, MRI = magnetic resonance imaging, NA = not available.

Among the previously reported *VPS13C*-related Parkinsonism cases with brain MRI findings (n = 11), 8 revealed symmetrical or asymmetrical lobar atrophic changes, and cerebellar cortex atrophy may be the earliest sign. Our study presents the longitudinal imaging data. The follow-up MRI demonstrated a progressive worsening of cortical atrophy as the disease advanced, which was paralleled by a cognitive decline evidenced by decreasing scores on the Mini-Mental State Examination and Montreal Cognitive Assessment. The extent of cortical and hippocampal atrophy may be associated with disease duration and cognitive function. Brain MRI may serve as a potential imaging marker of disease progression. However, further cohort studies are necessary. Despite the clinical heterogeneity, the pathology resembled that of DLB disease. Notably, tau-immunoreactive neurofibrillary tangles and neurites are observed in the brainstem, hippocampus, and cortex.^[1,16]^ The co-occurrence of α-synuclein and tau pathologies may contribute to the pathological and clinical heterogeneity of *VPS13C*-associated Parkinsonism.

*VPS13C* mutations exhibit high genetic diversity, with point mutations being the most common type, followed by splice-site mutations. Rare compound heterozygous and homozygous mutations in *VPS13C* have been identified as pathogenic. The 2 novel pathogenic variants (c.1699C > T, chr15: 62156504–62352664) identified in this study expand the known mutational spectrum of *VPS13C*-associated Parkinsonism. Literature review indicates that in China, the c.1291-4A > G mutation is frequently observed and is linked to the PD phenotype. Conversely, the c.2755G > T and c.10522C > T mutations are commonly found in Japanese cases and are correlated with the DLB phenotype. Interestingly, patients with a large homozygous *VPS13C* deletion have milder clinical manifestations and a normal cognitive status.^[2]^ There may be an association between genotype and phenotype.

However, we acknowledged that there were limitations in our present study. First, because the sample size of *VPS13C* gene related to Parkinsonism is relatively small, this leads to limitations in the clinical and phenotypic comparison of different *VPS13C* gene mutations. The clinical and genetic characteristics of *VPS13C* gene-related PD or DLB patients will be clarified in the future with ongoing case reports. Second, molecular neuroimaging such as DaT-SPECT was not performed. Third, since no functional experiments were performed in this study, the potential effects of the variants reported here should be examined in additional studies.

## 4. Conclusion

This study reports a case of EOPD caused by 2 novel variants of *VPS13C* and reviews previous reports. Significant clinical heterogeneity has been observed in *VPS13C*-related Parkinsonism. WES plays an important role in diagnosis and brain MRI may serve as a potential imaging marker for disease progression. This expands the genetic pedigree of the disease and increases clinicians’ understanding of the clinical and genetic characteristics of *VPS13C*-related Parkinsonism. Furthermore, the study of PD genetics provides invaluable insights into the disease’s pathophysiology and informs the development of disease-modifying therapies.

## Acknowledgments

We thank the patient and his family for their participation in this study.

## Author contributions

**Conceptualization:** Yanyan Jiang.

**Writing – original draft:** Yanyan Jiang.

**Writing – review & editing:** Chenghe Fan.

## References

[1] Lesage S Drouet V Majounie E. Loss of VPS13C function in autosomal-recessive Parkinsonism causes mitochondrial dysfunction and increases PINK1/parkin-dependent mitophagy. Am J Hum Genet. 2016;98:500–13. 26942284 10.1016/j.ajhg.2016.01.014 PMC4800038

[2] Darvish H Bravo P Tafakhori A. Identification of a large homozygous VPS13C deletion in a patient with early-onset Parkinsonism. Mov Disord. 2018;33:1968–70. 30452786 10.1002/mds.27516 PMC6309582

[3] Hopfner F Mueller SH Szymczak S. Rare variants in specific lysosomal genes are associated with Parkinson’s disease. Mov Disord. 2020;35:1245–8. 32267580 10.1002/mds.28037

[4] Rai SN Singh S Singh SK editors. Neurodegenerative Diseases: Translational Models, Mechanisms, and Therapeutics. 1st ed. CRC Press; 2025.

[5] Xu P Mancuso RI Leonzino M Zeiss CJ Krause DS De Camilli P. Impaired hematopoiesis and embryonic lethality at midgestation of mice lacking both lipid transfer proteins VPS13A and VPS13C. PLoS Biol. 2025;23:e3003393. 40956846 10.1371/journal.pbio.3003393 PMC12463328

[6] Schreglmann SR Houlden H. VPS13C-another hint at mitochondrial dysfunction in familial Parkinson’s disease. Mov Disord. 2016;31:1340. 27213732 10.1002/mds.26682

[7] Kumar N Leonzino M Hancock-Cerutti W. VPS13A and VPS13C are lipid transport proteins differentially localized at ER contact sites. J Cell Biol. 2018;217:3625–39. 30093493 10.1083/jcb.201807019 PMC6168267

[8] Hancock-Cerutti W Wu Z Xu P. ER-lysosome lipid transfer protein VPS13C/PARK23 prevents aberrant mtDNA-dependent STING signaling. J Cell Biol. 2022;221:e202106046. 35657605 10.1083/jcb.202106046 PMC9170524

[9] Schrӧder LF Peng W Gao G Wong YC Schwake M Krainc D. VPS13C regulates phospho-Rab10-mediated lysosomal function in human dopaminergic neurons. J Cell Biol. 2024;223:e202304042. 38358348 10.1083/jcb.202304042 PMC10868123

[10] Wang X Xu P Bentley-DeSousa A. The bridge-like lipid transport protein VPS13C/PARK23 mediates ER-lysosome contacts following lysosome damage. Nat Cell Biol. 2025;27:776–89. 40211074 10.1038/s41556-025-01653-6 PMC12081312

[11] Schormair B Kemlink D Mollenhauer B. Diagnostic exome sequencing in early-onset Parkinson’s disease confirms VPS13C as a rare cause of autosomal-recessive Parkinson’s disease. Clin Genet. 2018;93:603–12. 28862745 10.1111/cge.13124

[12] Kobayashi R Naruse H Koyama S. Familial dementia with Lewy bodies with VPS13C mutations. Parkinsonism Relat Disord. 2020;81:31–3. 33039764 10.1016/j.parkreldis.2020.10.008

[13] Harper L Barkhof F Fox NC Schott JM. Using visual rating to diagnose dementia: a critical evaluation of MRI atrophy scales. J Neurol Neurosurg Psychiatry. 2015;86:1225–33. 25872513 10.1136/jnnp-2014-310090

[14] Wittke C Petkovic S Dobricic V. Genotype-phenotype relations for the atypical Parkinsonism genes: MDSGene systematic review. Mov Disord. 2021;36:1499–510. 34396589 10.1002/mds.28517 PMC9070562

[15] Gu X Li C Chen Y. Mutation screening and burden analysis of VPS13C in Chinese patients with early-onset Parkinson’s disease. Neurobiol Aging. 2020;94:311.e1–4. 10.1016/j.neurobiolaging.2020.05.005 32507414

[16] Smolders S Philtjens S Crosiers D. Contribution of rare homozygous and compound heterozygous VPS13C missense mutations to dementia with Lewy bodies and Parkinson’s disease. Acta Neuropathol Commun. 2021;9:25. 33579389 10.1186/s40478-021-01121-w PMC7881566

[17] Monfrini E Spagnolo F Canesi M. VPS13C-associated Parkinson’s disease: Two novel cases and review of the literature. Parkinsonism Relat Disord. 2022;94:37–9. 34875562 10.1016/j.parkreldis.2021.11.031

